# Motor system modulation by transcranial alternating current stimulation: insights from functional MRI—a scoping review

**DOI:** 10.3389/fneur.2025.1684725

**Published:** 2025-10-31

**Authors:** Simon Han, Jason Luo, Ivy Tuong Van Vo, Won Kee Chang, Nam-Jong Paik, Ji Soo Choi, Won-Seok Kim

**Affiliations:** ^1^Department of Neurology, David Geffen School of Medicine, University of California, Los Angeles, Los Angeles, CA, United States; ^2^Department of Neurology, University of California, Los Angeles, Los Angeles, CA, United States; ^3^Department of Rehabilitation Medicine, Seoul National University College of Medicine, Seoul National University Bundang Hospital, Seongnam, Republic of Korea

**Keywords:** transcranial alternating current stimulation, motor system, functional magnetic resonance imaging, network, neuromodulation, stroke

## Abstract

Transcranial alternating current stimulation (tACS) is a non-invasive neuromodulation technique that delivers oscillatory currents to modulate endogenous brain rhythms. Frequency-specific effects on motor function have been reported, yet the neural mechanisms remain incompletely understood. This scoping review synthesizes functional MRI (fMRI) evidence on tACS-induced modulation of motor-related brain activity and connectivity in healthy individuals and patients with neurological conditions. A systematic search of the literature identified six eligible studies with a total of 108 participants, of whom 26 were individuals with chronic stroke. Stimulation frequencies ranged from 5 to 70 Hz, most often targeting the primary motor cortex. Gamma-band tACS (≥50 Hz) was generally associated with increased task-related activation and strengthened connectivity within sensorimotor networks in healthy participants, whereas alpha- and beta-band stimulation produced variable or region-specific effects. In the chronic stroke group, 10 Hz tACS enhanced localized activation, while 20 Hz tACS promoted broader network integration. These findings suggest that tACS may modulate motor networks in a frequency- and site-dependent manner, with preliminary implications for post-stroke rehabilitation. However, substantial heterogeneity in study design, stimulation parameters, and analysis approaches limits direct comparison across studies. Standardized protocols, larger clinical trials, and multimodal approaches integrating fMRI with electroencephalography are warranted to clarify underlying mechanisms and optimize tACS applications for motor recovery.

## 1 Introduction

Transcranial alternating current stimulation (tACS) is a promising non-invasive brain stimulation technique which uses scalp electrodes to deliver alternating electrical current to the brain. Studies have shown that tACS has the ability to modulate neural activity by altering the brain's endogenous oscillations, making it a useful tool in developing therapeutic interventions (1). One advantage of tACS is its ability to entrain specific neural rhythms and allow researchers to investigate frequency-dependent effects on the brain. Despite this potential, the underlying neural mechanisms of tACS remain poorly understood, especially as they relate to brain network connectivity and behavior in motor learning.

Motor performance is critical for daily life, being responsible for many essential functions. However, it is disrupted in neurological conditions such as stroke, Parkinson's disease, and traumatic brain injury (2–4). Motor impairments affect close to 80% of stroke survivors with many patients never fully regaining complete motor function even after intensive rehabilitation (5). Similarly, motor deficits such as bradykinesia are a hallmark characteristic of Parkinson's Disease, manifesting almost universally across patients. significantly lowering their quality of life (4). Furthermore, TBI can produce long-lasting disabilities, with 53% of mild TBI patients reporting functional deficits 12 months post-injury (2). Research indicates that even modest improvements in motor function can translate into increases in overall quality of life, making motor rehabilitation an important topic (3).

Recently, tACS has emerged as a potential neurorehabilitation method to enhance motor function. However, there is limited data showing its efficacy on large-scale brain networks. Functional magnetic resonance imaging (fMRI) is a neuroimaging tool for analyzing brain activity and functional connectivity by measuring hemodynamic changes over time. In particular, resting-state fMRI (rsfMRI) allows researchers to analyze the brain's intrinsic neural network dynamics via blood oxygen level dependent (BOLD) signals (6). Combining fMRI techniques with tACS can shed light on the neural mechanisms involved in tACS as well as the connection between behavioral motor outcomes and their functional neural correlates (7). However, to date, a comprehensive synthesis of studies combining tACS with fMRI in the context of motor function is lacking.

This scoping review systematically examines studies that combine tACS and fMRI in the context of motor function. We included studies conducted in both healthy individuals and clinical populations, particularly those with neurological conditions such as stroke. Specifically, this review aims to: (1) characterize the stimulation parameters and fMRI paradigms used; (2) summarize the reported effects of tACS on motor-related brain activation and connectivity in fMRI; (3) Identify methodological limitations and research gaps to guide future work in this field.

## 2 Methods

This review was conducted in accordance with PRISMA-ScR (Preferred Reporting Items for Systematic Reviews and Meta-Analyses extension for Scoping Reviews) guidelines (8).

### 2.1 Inclusion criteria

The inclusion and exclusion criteria were guided by the Population, Intervention, Comparison, and Outcome (PICO) framework. The population included human subjects. The intervention examined was tACS of varying frequencies. Comparisons were made between subjects receiving tACS with those receiving phase-synchronized and sham tACS. The primary outcome measured was motor learning which was assessed through fMRI-related benchmarks such as resting-state functional connectivity and task-related activity.

### 2.2 Exclusion criteria

Case reports, conference abstracts, duplicate studies, editorial opinions, letters, studies on oscillatory transcranial direct current stimulation (tDCS), protocol papers, review articles, studies on responsive neurostimulation, and studies on transcranial interferential stimulation were excluded.

### 2.3 Information source

A comprehensive search was conducted on October 20, 2023, across the Cochrane Library, EMBASE, and PubMed databases.

### 2.4 Search strategy

A systematic search was performed using both Medical Subject Headings (MeSH) terms and keyword-based queries to identify studies primarily focused on examining the effects of tACS on fMRI neuroimaging. A full description of the database-specific search strategies is provided in Supplementary material.

A total of 1,742 studies were identified: 144 from the Cochrane Library, 1,285 from EMBASE, and 313 from PubMed. After removing 418 duplicate records, 1,324 studies remained (Supplementary Figure 1).

### 2.5 Study screening

WSK and JC performed the title and abstract screening based on the inclusion and exclusion criteria. From the 36 studies retrieved for full-text evaluation, nine were excluded for the following reasons: (1) Two studies used transcranial electrical stimulation (tEC) instead of tACS. (2) Two studies provided only baseline fMRI data. (3) Two studies were non-interventional. (4) One study did not acquire fMRI data following tACS. (5) One study was a secondary analysis of another included study. (6) One study was a case report.

From the remaining studies, only those that investigated the effects of tACS on fMRI outcomes related to the motor domain were included which left six studies (Supplementary Figure 1).

### 2.6 Data extraction

Data were extracted from the six included studies by WSK and JC, encompassing the following components: (1) Participant Characteristics: Health status (healthy vs. stroke), sample size, age, sex, and intervention group. (2) tACS Parameters: Type of stimulator, target site, electrode position, frequency, frequency band, intensity, electrode surface area, and duration of stimulation, ramp-up & down, sham stimulation, and tACS modulation. (3) fMRI Parameters: Scanner type, magnetic field strength, number of channels in the MR coil, imaging method, repetition time, echo time (TE), flip angle (FA), voxel size, fMRI timing, and study design. (4) Results: Outcomes related to resting-state functional connectivity, task-related activity, and task-related effective connectivity.

### 2.7 Quality assessment

WSK and SH independently rated the risk of bias for each included study using the Cochrane Risk of Bias (RoB) tool (17). After independent assessments were completed, ratings were compared, and discrepancies were resolved. A visualization of the RoB assessment is provided in Supplementary Figure 2.

## 3 Results

Six studies including a total of *n* = 108 study subjects (*n* = 26 chronic stroke patients) investigating the effects of tACS on motor function and its neural correlates measured by fMRI were reviewed. All six studies delivered tACS non-invasively to cortical or cerebellar targets using surface electrodes (Table 1). Stimulation frequencies ranged from low theta (5 Hz) to high gamma (70 Hz), with the most common bands being alpha (10 Hz, *n* = 3 studies), beta (20 Hz, *n* = 4), and gamma (50 Hz, *n* = 2). Most studies, *n* = 5, targeted the primary motor cortex (M1), while *n* = 1 study targeted the cerebellum (Table 1). Electrode montages were guided by either 20 EEG systems or individualized hotspots identified through transcranial magnetic stimulation (TMS). Stimulation durations varied from brief task-aligned bursts (ex. 18 s in Moisa et al. (9) to 20 min in Chen et al. (10) and Yuan et al. (11) (Table 1). All six studies contained a sham control condition and were cross-over designs. A summary of the study designs can be found in Table 1.

**Table 1:** Summary of study design and tACS parameters.

**First author (year)**	**Study size**	**Age (mean ±SD)**	**Sex (M/F)**	**Study popu-lation**	**Interven-tion groups (*n*)**	**tACS stimulator brand**	**tACS target**	**tACS electrode position (10–20 system)**	**tACS fre-quency**	**tACS fre-quency (band)**	**tACS intensity (peak-to-peak)**	**Electrode surface area (cm^2^)**	**tACS dura-tion**	**Ramp-up and down**	**Sham stim-ulation**	**tACS modul-ation**
Bächinger (13)	*n =* 35	24.8 ± 4.1	10/10	Healthy	power-synchronized tACS: 20 phase-synchronized tACS: 20	NeuroConn	Bilateral M1	TMS spot (C3 and C4)-Oz	IAF at C3-C4	Alpha	1.5 mA	5 × 7	7 min	n/s	–	1 Hz amplitude envelope
Chen (10)	*n =* 13	61 ± 10	8/5	Stroke	10 Hz tACS: 13; 20 Hz tACS: 13; sham: 13	NeuroConn	Ipsilesional M1	C4-FP1	10 Hz, 20 Hz	Alpha, beta	1 mA	5 × 5	20 min	30 s	First 30 s	–
Moisa (9)	*n =* 20	24.1 ± 3.2	15/5	Healthy	20 Hz tACS: 20; 70 Hz tACS: 20; sham: 20	NeuroConn	Left M1	TMS spot (C3)-Lt shoulder	20 Hz, 70 Hz	Beta, gamma	1 mA	Active: 5 × 7; reference: 10 × 10	18 s	2 s	First 4 s (to 0.5 mA)	–
Weinrich (14)	*n =* 12	26 (range 21–42)	7/5	Healthy	20 Hz tACS: 12; 5 Hz tACS: 12; sham: 12	NeuroConn	Left M1	C3-FP2	20 Hz, 5 Hz	Beta, theta	1 mA	5 × 7	80 s × 4	10 s	First and last 10 s	–
Wessel (12)	*n =* 15	26.20 ± 3.34	8/7	Healthy	active: 15; sham: 15	NeuroConn	Left cerebellum	Active: 3 cm lateral to the inion (O1); Return: ipsilateral buccinator	50 Hz	Gamma	2 mA	5 × 5	20 min	2 s	30 s	–
Yuan (11)	*n =* 13	61 ± 10	8/5	Stroke	10 Hz tACS: 13; 20Hz tACS: 13; sham: 13	NeuroConn	Ipsilesional M1	C3-FP2	10 Hz, 20 Hz	Alpha, beta	1 mA	5 × 5	20 min	30 s	1 min	–

### 3.1 tACS on behavioral outcomes

Behavioral outcomes were evaluated in healthy participants in two studies using gamma tACS (>50 Hz). Moisa et al. (9) used beta (20 Hz), gamma (70 Hz), and sham tACS over the right M1 at the TMS-determined C3 hotspot responsible for eliciting twitch response in the right First Dorsal Interosseus (FDI) in 20 s blocks. Gamma tACS was able to increase movement velocity and acceleration compared to both the sham and beta tACS conditions, which elicited no significant difference in either velocity or acceleration. In comparison, Wessel et al. (12), the other study that examined gamma tACS revealed no improvements in skill acquisition or retention when the left cerebellum was stimulated at O1, 3 cm lateral to the inion. Instead, using linear mixed effects modelling, their findings showed that individual differences in baseline motor performance and short-interval intracortical inhibition during movement [SICI(move)] were the strongest predictors for training success, not stimulation (Table 1). Studies evaluating stroke patients did not report direct behavioral outcomes.

### 3.2 fMRI correlates of tACS

The effects of tACS on fMRI outcomes was investigated in all six studies, although their protocols varied (Table 2). Bächinger et al. (13) was the only study whose protocol used envelope tACS, a method in which the alpha frequency (IAF) was amplitude modulated in a slower 1 Hz envelope to mimic the natural cross frequency coupling, a phenomenon where a slower wave (e.g., delta) modulates the amplitude of faster rhythms (e.g., alpha). In this study, the researchers targeted the left and right sensorimotor cortices with the same envelope-tACS signal but altered the phase relationships between hemispheres to test two stimulation conditions; In the power-synchronized tACS condition, the 1 Hz amplitude envelopes were in-phase across hemispheres, while the underlying IAF signals were out of phase. In the phase-synchronized tACS condition, the IAF signals were in-phase, but the 1 Hz amplitude envelopes were out of phase. Voxelwise fMRI demonstrated that bilateral tACS application targeting the motor cortex led to a small but significant cluster of increased activity in the pre- and post-central gyri, particularly during power-synchronized stimulation. Electric field modeling confirmed peak stimulation in the postcentral gyrus extending toward premotor areas. Furthermore, power-synchronized tACS produced a 25% increase in sensorimotor network strength from baseline and a significant 20% increase compared to phase-synchronized tACS (*p* = 0.037). This increase in network strength persisted post stimulation as well, with power-synchronized aftereffects also significantly greater than phase-synchronized (*p* = 0.010). This suggests power-synchronized signals increase network connectivity, whereas phase-synchronized signals have minor effects.

**Table 2:** Summary of fMRI study parameters and main findings.

**Author (year)**	**MRI scanner brand**	**Magnetic field**	**Number of channels in MR coil**	**Imaging method**	**TR (ms)**	**TE (ms)**	**FA (°)**	**Voxel size (mm3)**	**fMRI timing**	**fMRI design**	**Main outcomes**
Bächinger et al. (13)	Philips Achieva	3 T	8	n/s	2,100	30	79	3 × 3 × 3	Baseline, online, offline	Resting-state	Power -synchronized tACS strengthens sensorimotor networks
Chen et al. (10)	Philips Achieva	3 T	8	GE-EPI	2,000	30	70	2.8 × 2.8 × 3.5	Baseline, online, offline	Resting-state	β tACS facilitates local segregation and whole brain integration; α: facilitates whole brain segregation
Moisa et al. (9)	Philips Achieva	3 T	8	Echo-planar imaging	3,000	35	79	2.5 × 2.5 × 2.5	Online	Task (movement initiation, grip control)	γ tACS improved motor function, increased BOLD in M1 and S1, and decreased dmPFC activity.
Weinrich et al. (14)	Siemens Verio	3 T	32	Multi-band EPI	1,300	40	n/s	2 × 2 × 2	Online	Resting-state	β tACS uncoupled M1 from broader motor network.
Wessel et al. (12)	Siemens Prisma	3 T	n/s	Echo-planar imaging	1,000	32	50	2 × 2 × 2	Online, offline	Resting-state	Reduced functional connectivity between PPC and right caudate
Yuan et al. (11)	Philips Achieva	3 T	8	GE-EPI	2,000	30	70	2.8 × 2.8 × 3.5	Baseline, online, offline	Resting-state, task (maximal hand grasp)	α tACS increased activation in ipsilesional PreCG; β tACS enhanced post-stim connectivity in contralateral OP and ACC

n/s, not specified; TR, Repetition Time; TE, Echo Time; FA, Functional Anisotropy; M1, Primary Motor Cortex; S1, Primary Somatosensory Cortex; dmPFC, Dorsomedial Prefrontal Cortex; PPC, Posterior Parietal Cortex; PreCG, Precentral Gyrus; OP, Operculum; ACC, Anterior Cingulate Cortex.

Moisa et al. (9) found that gamma tACS which improved motor outcomes was significantly positively correlated with increased BOLD activity in the M1 and primary somatosensory cortex (S1). However, this stimulation was also associated with a decrease in brain activity in the dorsomedial prefrontal cortex (dmPFC) during movement initiation and an increase in dmPFC activity during the grip task. The decrease in dmPFC activity was interpreted as a remote network effect rather than direct stimulation because the simulated electric field at the dmPFC was measured to be near zero. The authors suggest this decrease in dmPFC activity may act as a compensatory mechanism, and that the reduced top-down control may allow for increased motor execution under gamma tACS. Moreover, the authors conducted a psychophysiological interaction (PPI) analysis and found that gamma tACS enhanced functional connectivity between the dmPFC and motor regions including the stimulated M1, supplementary motor area Wessel et al. (12), bilateral thalamus, and left putamen, suggesting that stimulation modulates motor-related networks beyond the stimulation site.

Weinrich et al. (14) and Wessel et al. (12) used rsfMRI to examine tACS effects on motor-related network functional connectivity in healthy participants, differing in targets, frequencies, and outcomes. Weinrich applied 20 Hz and 5 Hz tACS over the left M1 and examined connectivity within motor networks. While 20 Hz stimulation did not significantly increase motor network connectivity, it selectively uncoupled M1 from the broader motor network, disrupting its typical correlation with network strength. The default mode network was unaffected, suggesting a frequency and network specific desynchronization effect. In contrast, Wessel et al. (12) discovered that 50 Hz tACS over the left cerebellum aimed to modulate motor skill learning reduced functional connectivity between the posterior parietal cortex (PPC) and the right caudate. This dissociation between neural and behavioral outcomes suggests that cerebellar gamma tACS can induce measurable network changes, even in the absence of performance gains. These studies demonstrate that tACS effects are highly dependent on target region and frequency, with Weinrich's results emphasizing cortical decoupling through beta stimulation, and Wessel's pointing to subcortical network modulation through cerebellar gamma tACS.

Two studies assessed the impacts of tACS on chronic stroke patients. Chen et al. (10) and Yuan et al. (11) specifically examined the effects of 10 Hz and 20 Hz tACS over the ipsilesional M1 in a sham and stroke group, using similar stimulation targets but yielding distinct neural outcomes. However, their designs differed in fMRI methodology and analytical focus. Chen used rsfMRI combined with graph theory analysis to assess changes in network organization, reporting that 20 Hz tACS increased clustering coefficient and local efficiency within motor-related regions, suggesting enhanced segregation and integration. 10 Hz tACS promoted segregation, while 20 Hz promoted integration. In contrast, Yuan combined resting-state and task-based fMRI to examine motor-related activation during a maximal hand grasp task. Yuan found that 10 Hz tACS increased activation in the ipsilesional precentral gyrus during paretic hand movement and decreased contralesional central connectivity during stimulation, whereas 20 Hz tACS enhanced post-stimulation connectivity in the contralesional operculum and anterior cingulate. Only Yuan assessed task-specific brain activation and linked it to motor output, while Chen focused on resting-state dynamics. Together, these studies suggest that both frequency and analysis approach influence outcomes with 20 Hz tACS showing broader effects on global network integration, and 10 Hz tACS supporting localized motor recovery processes.

## 4 Discussion

Ultimately, our scoping review identified six studies that utilized concurrent tACS and fMRI to assess modulation of brain connectivity as it relates to motor function. While there was considerable heterogeneity in study parameters, tACS interventions, and imaging designs, several crucial themes emerged.

First, gamma-band tACS applied over motor locations in the brain (e.g., M1 or SMA) was associated with enhanced task-related activation and connectivity in sensorimotor networks beyond the immediate stimulation site including regions (e.g., bilateral thalamus and left putamen), especially in healthy participants (1, 9, 12, 14). In contrast, alpha or beta-band stimulation showed more unpredictable effects, particularly in resting-state paradigms. For example, Bächinger et al. demonstrated frequency-specific modulation of resting-state fMRI connectivity between bilateral motor regions during alpha-tACS, while Chen et al. and Yuan et al. showed altered network segregation or integration in stroke survivors depending on stimulation frequency. Based on these findings, the data suggests that gamma-band application is more likely to be responsible for motor performance (1, 9) whereas alpha and beta-band stimulation may have a role in natural inhibition of motor control through attenuation and suppression (4, 13, 14). The variability of these outcomes in movement related changes highlights the important need to understand the exact mechanisms that each frequency plays on the brain's networks (1).

Despite these promising results, heterogeneity in study designs remains a significant barrier to fully synthesizing these findings and limits researchers from creating generalizable conclusions (1, 7). For example, most tACS–fMRI studies to date, including those reviewed here, have been conducted in healthy individuals, often using high-frequency (gamma) stimulation, which has been associated with transient improvements in motor performance. In contrast, the limited studies in stroke patients have primarily applied lower frequencies (alpha and beta), which appear to support network reorganization processes rather than immediate behavioral gains. The inclusion of data from healthy participants remains crucial for providing mechanistic insights into frequency-dependent modulation and for generating hypotheses for clinical translation. Nevertheless, due to pathological alterations in neural networks after stroke, direct translation should be made with caution, as the optimal stimulation frequencies for recovery may differ from those effective in healthy brains.

In addition, there is significant variability in electrode montage (e.g., M1-SMA, M1-Cz, cerebellum), task condition (resting-state vs. motor task), and outcome metrics (functional connectivity, regional activation, graph metrics). To accurately and correctly interpret the data from these different studies, heterogeneity must be limited. It is imperative to extend the current literature through similarly designed studies that apply similar frequencies of tACS in similar areas during standardized subject tasks to both healthy and stroke patients (8). Thus, this large range of variability limits our ability to generalize conclusions based on these results and complicates the identification of optimal stimulation parameters.

Moreover, technical challenges of combining tACS with fMRI were noted. MR-compatible stimulation equipment, synchronization methods, and artifact suppression techniques (e.g., adaptive filtering, post-processing corrections) are not yet standardized across studies which definitely could have impacted BOLD signal interpretation (1, 7, 13).

From a translational perspective, the studies on clinical populations such as chronic stroke patients provide preliminary insights into how tACS may affect impaired motor networks (10, 11). However, currently, the majority of studies investigating the behavioral impacts of tACS are mainly assessed in healthy patients. Behavioral outcomes in stroke patients have been less systematically reported, and the two included fMRI studies with stroke patients did not provide direct behavioral data. Thus, there are currently no studies directly linking fMRI changes and behavioral outcomes in stroke patients, limiting the promising results of non-invasive brain stimulation evident in healthy patients (2, 3, 5). Nevertheless, only a few studies have examined behavioral effects of tACS in stroke patients, and their findings remain inconsistent. For instance, Kitatani et al. (15) demonstrated that gait-synchronized oscillatory brain stimulation over the ipsilesional primary motor cortex foot area, a tACS-like approach, enhanced β-band intermuscular coherence between ankle muscles and improved gait symmetry in chronic stroke survivors, whereas Grigutsch et al. (16) observed that θ-γ-tACS could even diminish motor learning efficiency in stroke survivors. These mixed and limited findings indicate that the behavioral impact of tACS in patients with pathologically altered neural networks remains uncertain, and the small number of available studies makes it difficult to draw firm conclusions about its clinical benefits. Therefore, further research is needed to concurrently assess neurophysiological changes, such as fMRI-based network dynamics, alongside behavioral outcomes in stroke patients, ideally through longitudinal studies applying repeated tACS sessions to elucidate the causal relationships between neural modulation and functional recovery.

Currently, many researchers are studying the effects of tACS through EEG or behavioral outcomes that are observed and quantified. EEG, for instance, provides great temporal resolution for electrical brain activity, allowing scientists to gain instant insight into how the brain is changing in real time based on applied tACS (1, 7). However, EEG lacks the ability to localize brain oscillation activation, limiting our ability to infer the mechanism of change. Significantly, fMRI can be used to complement EEG findings by providing high quality spatial resolution and connectivity mapping across brain regions, allowing for a multimodal biomarker approach in neurorehabilitation (6, 13). Further research should pursue standardized stimulation protocols, harmonized imaging pipelines, and simultaneous EEG-fMRI approaches to deepen our understanding of tACS-induced neuromodulation (1, 7).

### 4.1 Future directions

Currently, tACS appears to be a promising intervention capable of translating modulation of neural networks into clinical improvements. However, further research is required to clarify the underlying mechanisms and optimize stimulation protocols for different patient population. Specifically, investigators should focus on tailoring stimulation frequency and montage not only to an individual's baseline network characteristics but also to dynamic state changes that occur during task performance or training, with consideration of closed-loop stimulation paradigms that adapt parameters in real time. In addition, future studies should focus on employing multimodal approaches that include combining tACS with simultaneous EEG-fMRI to link temporal and spatial neural markers, establishing multimodal biomarkers of tACS-induced neural modulation. It would also strengthen the current evidence by conducting longitudinal studies employing multi-session or adaptive tACS protocols to investigate sustained effects on motor recovery and network reorganization beyond the immediate changes observed after single sessions. Lastly, future studies are needed to embed tACS within rehabilitation training or other recovery paradigms (e.g., task-based motor learning) to investigate potential synergistic effects on clinical outcomes.
