# Supplementary material for: Motor system modulation by transcranial alternating current stimulation: insights from functional MRI—a scoping review
Source: Front Neurol. 2025 Oct 31;16:1684725. doi: 10.3389/fneur.2025.1684725 (PMC12615180; doi:10.3389/fneur.2025.1684725)
Supplement: Supplementary file 1 [file Table_1.docx]

# **Supplementary Table 1.** Search Strategy.

| *For Cochrane Library* |
| --- |
| 1. [mh "Transcranial Direct Current Stimulation"] 2. Transcranial Alternating Current Stimulation":ti,ab,kw OR "Transcranial Electrical Stimulation":ti,ab,kw OR "Electrical Stimulation, Transcranial":ti,ab,kw OR "Electrical Stimulations, Transcranial":ti,ab,kw OR "Stimulation, Transcranial Electrical":ti,ab,kw OR "Stimulations, Transcranial Electrical":ti,ab,kw OR "Transcranial Electrical Stimulations":ti,ab,kw OR "Repetitive Transcranial Electrical Stimulation":ti,ab,kw OR "tACS":ti,ab,kw OR "transcranial pulsed current stimulation":ti,ab,kw OR "transcranial individual neurodynamics stimulation":ti,ab,kw OR "Transcranial alternate current stimulation”:ti,ab,kw OR “Transcranial alternative current stimulation”:ti,ab,kw OR “Transcranial alternation current stimulation”:ti,ab,kw OR “Transcranial oscillation stimulation”:ti,ab,kw OR “Transcranial oscillating stimulation”:ti,ab,kw OR “Transcranial oscillate stimluation”:ti,ab,kw OR “Transcranial oscillatory stimulation”:ti,ab,kw OR “oscillatory stimluation, Transcranial":ti,ab,kw 3. #1 OR #2 4. “functional magnetic resonance imaging” OR “fMRI” OR “functional MRI” OR “functional connectivity MRI” OR "fcMRI" 5. #3 AND #4 |
| *For EMBASE* |
| 1. "transcranial direct current stimulation"/exp 2. "Transcranial Alternating Current Stimulation":ti,ab,kw,de OR "Transcranial Electrical Stimulation":ti,ab,kw,de OR "Electrical Stimulation, Transcranial":ti,ab,kw,de OR "Electrical Stimulations, Transcranial":ti,ab,kw,de OR "Stimulation, Transcranial Electrical":ti,ab,kw,de OR "Stimulations, Transcranial Electrical":ti,ab,kw,de OR "Transcranial Electrical Stimulations":ti,ab,kw,de OR "Repetitive Transcranial Electrical Stimulation":ti,ab,kw,de OR "tACS":ti,ab,kw,de OR "transcranial pulsed current stimulation":ti,ab,kw,de OR "transcranial individual neurodynamics stimulation":ti,ab,kw,de OR "Transcranial alternate current stimulation”:ti,ab,kw,de OR “Transcranial alternative current stimulation”:ti,ab,kw,de OR “Transcranial alternation current stimulation”:ti,ab,kw,de OR “Transcranial oscillation stimulation”:ti,ab,kw,de OR “Transcranial oscillating stimulation”:ti,ab,kw,de OR “Transcranial oscillate stimluation”:ti,ab,kw,de OR “Transcranial oscillatory stimulation”:ti,ab,kw,de OR “oscillatory stimluation, Transcranial":ti,ab,kw,de 3. #1 OR #2 4. “functional magnetic resonance imaging” OR “fMRI” OR “functional MRI” OR “functional connectivity MRI” OR "fcMRI" 5. #3 AND #4   *For PubMed* |
| 1. "Transcranial Direct Current Stimulation"[Mesh] 2. "Transcranial Alternating Current Stimulation"[TW] OR "Transcranial Electrical Stimulation"[TW] OR "Electrical Stimulation, Transcranial"[TW] OR "Electrical Stimulations, Transcranial"[TW] OR "Stimulation, Transcranial Electrical"[TW] OR "Stimulations, Transcranial Electrical"[TW] OR "Transcranial Electrical Stimulations"[TW] OR "Repetitive Transcranial Electrical Stimulation"[TW] OR "tACS"[TW] OR "transcranial pulsed current stimulation"[TW] OR "transcranial individual neurodynamics stimulation"[TW] OR "Transcranial alternate current stimulation”[TW] OR “Transcranial alternative current stimulation”[TW] OR “Transcranial alternation current stimulation”[TW] OR “Transcranial oscillation stimulation”[TW] OR “Transcranial oscillating stimulation”[TW] OR “Transcranial oscillate stimluation”[TW] OR “Transcranial oscillatory stimulation”[TW] OR “oscillatory stimluation, Transcranial"[TW] 3. #1 OR #2 4. “functional magnetic resonance imaging” OR “fMRI” OR “functional MRI” OR “functional connectivity MRI” OR "fcMRI"   #3 AND #4 |
