# Supplementary figures and images for: Motor system modulation by transcranial alternating current stimulation: insights from functional MRI—a scoping review
Source: Front Neurol. 2025 Oct 31;16:1684725. doi: 10.3389/fneur.2025.1684725 (PMC12615180; doi:10.3389/fneur.2025.1684725)

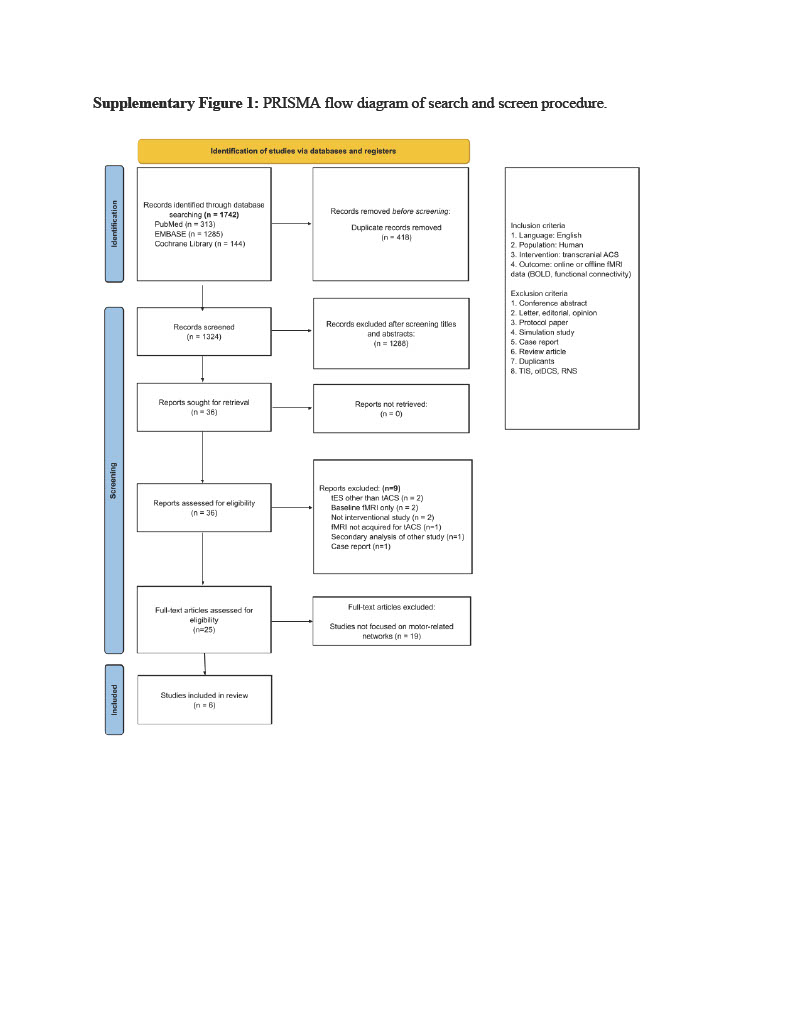

Supplement: Supplementary file 2 [file Image_1.jpeg]

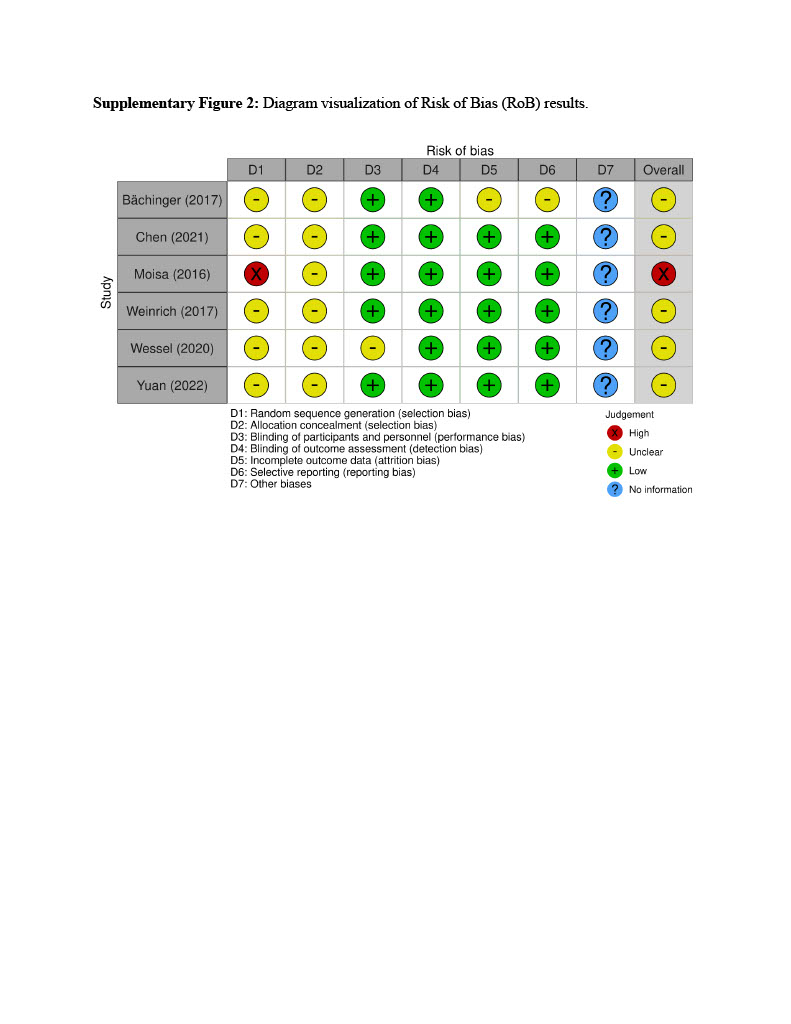

Supplement: Supplementary file 3 [file Image_2.jpeg]
